# Genetic profiling for *Anaplasma* and *Ehrlichia* species in ticks collected in the Eastern Cape Province of South Africa

**DOI:** 10.1186/s12866-017-0955-0

**Published:** 2017-02-27

**Authors:** Benson C. Iweriebor, Elia J. Mmbaga, Abiodun Adegborioye, Aboi Igwaran, Larry C. Obi, Anthony I. Okoh

**Affiliations:** 1 SAMRC Microbial Water Quality Monitoring Centre, University of Fort Hare, Private Bag X1314, Alice, 5700 Eastern Cape South Africa; 2 Applied and Environmental Microbiology Research Group (AEMREG), Department of Biochemistry and Microbiology, University of Fort Hare, Private Bag X1314, Alice, 5700 Eastern Cape Province South Africa; 3 The Muhimbili University of Health and Allied Sciences (MUHAS), Dar es Salaam, Tanzania; 4 Academic and Research Division, University of Fort Hare, Private Bag X1314, Alice, 5700 Eastern Cape Province South Africa

**Keywords:** Anaplasmosis, Ehrlichiosis, South Africa, Tick-borne

## Abstract

**Background:**

*Anaplasma* and *Ehrlichia* are emerging tick-borne pathogens that cause anaplasmosis and ehrlichiosis in humans and other animals worldwide. Infections caused by these pathogens are deadly if left untreated. There has been relatively no systematic survey of these pathogens among ticks in South Africa, thus necessitating this study. The presence of *Anaplasma* and *Ehrlichia* species were demonstrated by PCR in ticks collected from domestic ruminants at some selected communities in the Eastern Cape of South Africa. The ticks were identified by morphological characteristics and thereafter processed to extract bacterial DNA, which was analyzed for the presence of genetic materials of *Anaplasma* and *Ehrlichia*.

**Results:**

Three genera of ticks comprising five species were identified. The screening yielded 16 positive genetic materials that were phylogenetically related to *Ehrlichia* sequences obtained from GenBank, while no positive result was obtained for *Anaplasma*. The obtained *Ehrlichia* sequences were closely related to *E. chaffeensis, E. canis*, *E. muris* and the incompletely described *Ehrlichia* sp. UFMG-EV and *Ehrlichia* sp. UFMT.

**Conclusion:**

The findings showed that ticks in the studied areas were infected with *Ehrlichia* spp. and that the possibility of transmission to humans who might be tick infested is high.

## Background

Ticks are well-known, excellent vectors for a variety of microorganisms, many of which are agents of emerging tick-borne zoonotic diseases [1]. Besides mosquitoes, ticks are the second-most notorious vector of human diseases; with more than 800 species of these obligate hematophagous parasites known to infest humans and animals globally [2]. The need to ingest a blood meal in order to molt to their next developmental stage makes ticks an excellent disease vector in both humans and animals.

Numerous bacterial pathogens of vertebrates, including *Anaplasma* spp. and *Ehrlichia* spp., are transmitted by ticks, and both genera contain obligate intracellular Gram-negative parasites, which are found in membrane-bound structures or vacuoles within the cytoplasm of the host cells [3–6]. There are four pathogens of ruminants in the genus *Anaplasma*: *A. marginale, A. centrale, A. bovis* and *A. ovis;* and in addition, there is *A. phagocytophilum,* which infects a variety of hosts, including humans and other animals, and *A. platys,* which infects dogs [7].

The genus *Ehrlichia* consists of *E. chaffeensis, E. canis, E. ewingii, E. muris* and *E. ruminantium,* all of which are capable of causing infections in both humans and domestic animals, consequently resulting in huge economic losses globally [7–11]. *Ehrlichia muris* is thought to be zoonotic and infects monocytes in rodents, whereas *E. ruminantium* is the etiologic agent of heartwater in ruminants and is not believed to be zoonotic. However, several people in South Africa have reportedly been infected, as it has been detected in the blood of HIV-infected patients [7].

Ehrlichiosis and anaplasmosis are severe, feverish tick-borne illnesses arising from infections attributed to many species of the genera *Ehrlichia* and *Anaplasma* of the family Anaplasmataceae. In the United States, human monocytotropic ehrlichiosis is regarded as one of the most prevalent and deadly tick-borne diseases [5] and they are beginning to emerge in other parts of the world. The intricate relationship between animal reservoirs, tick vectors and humans could result in increased frequency in the diagnoses of ehrlichiosis and anaplasmosis as human infections. Human ehrlichiosis is an emerging zoonotic infection that causes disease symptoms ranging from a mild feverish illness to an acute disease characterized by multiorgan system failure, while human granulocytotropic anaplasmosis (HGA) caused by *A. phagocytophilum* is characterized by an acute febrile illness [12, 13].

In South Africa, there is little or no information on the prevalence of tick-borne diseases among the populace, as studies in this regard are scarce. Because of the semi-arid nature of its vegetation, South Africa is known for animal rearing. Ticks are predominantly found in areas where the density of wild animals and birds is high. South Africa has a very high diversity of wild animals in many game reserves that share boundaries with rural communities. Virtually every rural community keeps domestic animals within their homesteads; and these animals, like their wild counterparts, are often infested with various tick species, which could harbour a variety of tick-borne pathogens. Tick-borne diseases most often present as fever and could easily be misdiagnosed by clinicians. There are numerous reports of travelers who have returned home from South Africa feeling sick with fever and were later diagnosed with tick-borne illnesses [14, 15].

The risk of tick-borne diseases is an important concern for many professionals, including farmers, forestry workers, military personnel and others who regularly engage in outdoor work; likewise, tourists who visit tick-infested areas and woodlands are also at risk [5, 16]. The Eastern Cape of South Africa is predominantly rural; containing many communities whose primary means of livelihood is animal rearing. These animals are kept near human dwellings and freely range within and between communities. Freely roaming animals in communities has human health implications, as zoonotic pathogens in infected animals can be easily transmitted to humans: A single bite from an infected tick could result in disease transmission. Data on the prevalence of any emerging pathogens are necessary for tracking their spread and possibly controlling their known vectors. The main objective of this study was to genetically profile the presence of *Anaplasma* spp. and *Ehrlichia* spp., which are the two pathogenic genera in the family Anaplasmataceae in ticks samples collected from domesticated animals that range freely within human dwellings in the Chris Hani and Amatole District Municipalities in Eastern Cape Province, South Africa.

## Methods

Ethical clearance was obtained from the University of Fort Hare Ethics Committee before the commencement of the study, while permission was obtained from farmers to collect ticks from their animals with the help of veterinary personnel and animal health technicians in charge of treating the animals.

### Sample collection

The collection of ticks was performed between March and June 2016 from domesticated ruminants (cattle, sheep and goats) and horses from Cofimvaba, with the geographical coordinates 32° 0′ 9″S 27° 34′ 50″E; Chemizile in the Chris Hani District Municipality, with the coordinates 32° 10′ 0″S 26° 49′ 0″E; and Alice in the Amatole District Municipality, with the coordinates 32° 47′S 26° 50′E. These study sites were chosen because they are primarily known for animal husbandry within Eastern Cape Province. Tick collection was arbitrarily conducted based on the availability of domestic animals, but efforts were made to obtain a widespread representative sample within the different animal species included in the study. Tick collection was carried out with the help of animal health technicians from the South African Department of Agriculture, Forestry and Fisheries (DAFF). University of Fort Hare Animal Ethics Committee regulations were strictly adhered to in the handling of the animals.

Ticks were collected into 50 mL Nalgene tubes containing 70% ethanol and transported to the Applied and Environmental Microbiology Research Group in the Microbiology Department at the University of Fort Hare. During sampling, care was taken to ensure that ticks collected from each animal were placed in separate tubes and properly labelled.

### Tick identification and bacterial DNA extraction

Following identification to species based on morphological criteria previously outlined by [17], the ticks were washed in sterile distilled water and chopped with a sterile blade in a Petri dish containing lysis buffer with proteinase K. Individual engorged ticks were extracted separately, while pools of 10 or more non-engorged ticks were extracted together according to the animal, place of collection and tick species delineation. Each chopped tick or pool was mechanically triturated in 10 mM Tris, 1 mM EDTA, 100 μg proteinase K per mL, and 0.5% sodium dodecyl sulfate lysing buffer, incubated at 60 °C for 1 h, and then centrifuged at 5000 rpm for 5 min;. The supernatants were then collected in a clean 2-mL microcentrifuge tube, after which DNA extraction was performed using the Zymo Research Quick-DNA Universal Kit (Zymo Research, Madison, WI, USA) according to the manufacturer’s instructions.

### Detection of *Anaplasma* spp. and *Ehrlichia* spp. in ticks

The presence of *Anaplasma* spp. and *Ehrlichia* spp. in the ticks were detected following the methods previously described by Sun et al. [18]. Briefly, we targeted the 16S RNA of *Anaplasma* spp. and the genus-specific disulfide bond formation protein (*dsb*A) gene of *Ehrlichia* spp. with the following primer pairs: ANA F 5′-GCAAGTCGAACGGATTATTC-3′ and ANAR 5′-TTCCGTTAAGAAGGATCTAATCTCC-3′ to generate a 932 bp PCR fragment, and EHL dsb-330 5′-GATGATGTCTGAAGATATGAAACAAAT-3′ and EHL dsb-728 5′-CTGCTCGTCTATTTTACTTCTTAAAGT-3′ to generate 409 bp fragments of *Anaplasma* 16S RNA and *Ehrlichia dsb*A genes, respectively. PCRs were performed in a 25-μL reaction mixture containing 12.5 μL of the master mix, 1 μL of 10 pMol for each of the forward and reverse primers, 5.5 μL of RNase nuclease-free water, and 5 μL of DNA template. The cycling conditions were as follows: an initial heating block at 94 °C for 3 min, followed by 35 cycles of denaturation at 93 °C for 30 s, then annealing at 50 °C for *Anaplasma* and 47 °C for *Ehrlichia* with an elongation at 72 °C for 1 min and a final elongation at 72 °C for 5 min. PCR amplification was verified in 1% gel electrophoreses stained with ethidium bromide, electrophoresed at 120 volts for 45 mins in 0.5X TBE buffer, visualized under a UV transilluminator and then photographed.

### Sequencing, sequence editing, and compilation

Positive amplicons were sequenced on both strands, and generated sequences were edited with Geneious bioinformatics tools (Biomatters Ltd, Auckland, NZ) and analyzed phylogenetically.

Automated population-based sequencing was performed on both strands of bacterial DNA with the forward and reverse primers that were used to amplify the target region of the *dsb*A of *Ehrlichia* spp. was detected using the dideoxynucleotide chain termination approach on an ABI Prism DNA genetic analyzer (ABI Prism 310, Applied Biosystems, Foster City, CA) while no sample was positive for *Anaplasma* spp. Forward and reverse nucleotide sequences were assembled and edited with the Geneious programme in the R9 software, version 9.1.5 [19].

### Blast search and phylogenetic analysis

The sequences data obtained after editing were analyzed for homologies with other sequences in GenBank with the BLAST program (http://blast.ncbi.nlm.nih.gov), with the parameters set on highly similar sequences and Ehrlichiaceae chosen as the organism option. Sequences with a percentage similarity above 95% were downloaded for phylogenetic tree analysis. Sequences generated in this study were aligned and compared with these GenBank reference sequences representing the different species of the *Ehrlichia*-partial *dsb*A gene. The sequences were then manually edited with the Geneious 9.1.5 align/assemble software, and gaps were removed from the final alignments. Neighbour-joining phylogenetic tree was constructed using the tree builder. In order to probe the statistical robustness of the tree, bootstrapping was performed with 1000 replicates.

### Nucleotide sequence accession numbers

Representative sequences obtained from this study have been deposited in the GenBank under the following accession numbers: **KX922864 – KX922879**.

## Results

A total of 760 adult hard ticks were collected in this study. These ticks belonged to three genera, *Rhipicephalus, Amblyomma* and *Hyalomma*, comprising five species. The most-to-least collected species were *Rhipicephalus eversti eversti* (360 adults), followed by *R. sanguineus* s.l. (180 adults), *R. appendiculatus* (120 adults), *Amblyomma hebraeum* (50 adults) and *Hyalomma marginatum rufipes* (50 adults). Similarity in the diversity of collected ticks was detected at each site except from the site with horses, where no tick was recovered. The species, number and prevalence of *Ehrlichia* in hard ticks at each collection site are shown in Table 1.

**Table 1:** Proportion and distribution of collected tick species and the prevalence of *Ehrlichia* spp.

Animal/Location	Tick species	Developmental stage	Number	Positive for *Ehrlichia* spp.
Cattle	*R. eversti eversti*	Adult	160	n/d
	*R. sanguineus* s.l.	Adult	70	3 (E2, E12, E15)
	*R. appendiculatus*	Adult	50	2 (E1, E8)
	*Rhipicephalus* spp.	Nymph	0	n/a
	*A. hebraeum*	Adult	25	3 (E10, E7, E13)
	*H. marginatum rufipes*	Adult	8	n/d
Sheep	*R. eversti eversti*	Adult	140	1 (E11)
	*R. sanguineus* s.l.	Adult	50	2 (E5, E14)
	*R. appendiculatus*	Adult	30	1 (E17)
	*Rhipicephalus* spp.	Nymph	0	n/a
	*A. hebraeum*	Adult	15	4 (E3, E4, E16, E9)
	*H. marginatum rufipes*	Adult	12	n/d
Goats	*R. eversti eversti*	Adult	60	n/d
	*R. sanguineus* s.l.	Adult	60	n/d
	*R. appendiculatus*	Adult	40	n/d
	*Rhipicephalus* spp.	Nymph	0	n/a
	*A. hebraeum*	Adult	10	n/d
	*H. marginatum rufipes*	Adult	30	n/d
Horse	*R. eversti eversti*	Adult	0	n/a
	*R. sanguineus* s.l.	Adult	0	n/a
	*R. appendiculatus*	Adult	0	n/a
	*Rhipicephalus* spp.	Nymph	0	n/a
	*A. hebraeum*	Adult	0	n/a
	*H. marginatum rufipes*	Adult	0	n/a

Key: *n/a* not available, *n/d* not detected

### Molecular detection of *Ehrlichia* spp. in ticks

**Fig. 1:**
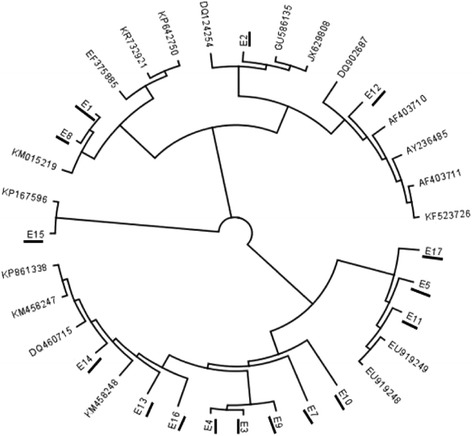
Phylogenetic relationship of various *Ehrlichia* spp. based on the nucleotide sequences of the genus-specific disulfide bond formation protein (*dsb*A) gene of *Ehrlichia* spp. The underlined are the *Ehrlichia* spp. sequences detected in this study. All the detected sequences in this study clustered phylogenetically with reference *Ehrlichia* spp. *dsb*A gene sequences obtained from the GenBank. The tree was drawn with Geneious version 9.1.5, created by Biomatters and available from http://www.geneious.com, Kearse et al., [19]

A total of 16 positive samples were obtained from the 760 ticks analyzed for the presence of *Anaplasma* and *Ehrlichia* genetic materials (DNA) using primer pairs specific to the thio-oxidoreductase protein gene (*dsb*A) of the *Ehrlichia* genus and 16S RNA gene-specific primers of *Anaplasma*. The identified positive samples were all of *Ehrlichia;* none were positive for *Anaplasma*. The samples identified from the respective tick species analyzed are shown in Table 1. A homology search for the obtained sequences from PCR data showed that they had a high sequence similarity of above 95% with homologous *dsb*A of other *Ehrlichia* sequences in GenBank. Sequence E8 had a 98% similarity to *Ehrlichia* sp. UFMG-EV (**JX629808**) and *Ehrlichia* sp. UFMT (**KT970783**), while E7 was 96% similar to *E. canis* (**KU534872**); likewise, E15 was 98% similar to *E. canis* (**DQ124254, KR732921**). Other sequences that demonstrated a high degree of similarity (98%) with *E. canis* sequences were E9, E12, E14, E1 and E2 (**KU534892**). E11 was 100% similar to *E. canis* (**KR732921, KP167596,** and **KU534872**). The other remaining sequences (E13, E4, E16, E7, E10, E3) were more than 97% similar to *E. chaffeensis* (**KM458248**), while sequences E17, E5 and E11 were 99% homologous to *E. muris* (**E919249, EU919248**). Phylogenetic analysis of the derived sequences was performed with these *Ehrlichia dsb*A GenBank reference sequences after an alignment was made via Geneious’ alignment editor and tree builder, both included in the software Biomatters Ltd [19]. In the constructed phylogenetic tree, the sequences obtained in this study clustered with reference sequences of *Ehrlichia* spp. obtained from GenBank homology search using BLAST (http://blast.ncbi.nlm.nih.gov). Sequences E5, E11 and E17 clustered with *E. muris* with accession numbers **EU919249** and **EU919248**, while sequences E10, E7, E9, E3 and E4 clustered close by; E16, E13 and E14 clustered with *E. chaffeensis* (**KMN458248**). Sequence E15 clustered phylogenetically with *E. canis* (**KP167596**); likewise, E2 and E12 clustered with **DQ124254** and **DQ902687**, respectively. E1 and E8 sequences clustered with *Ehrlichia* sp. UFMG-EV *minasensis* (**KM015219**), as shown in the phylogenetic tree (Fig. 1).

## Discussion

Members of the genus *Ehrlichia,* the causative agent of human monocytotrophic ehrlichiosis (HME), are becoming recognized as emerging tick-borne pathogens worldwide. *Ehrlichia* spp. are naturally transmitted by Ixodidae and are maintained between the ticks and wild or domestic animal reservoir hosts [20–22]. A total of 760 ticks were collected and analysed for the presence of *Anaplasma* and *Ehrlichia* spp. *Anaplasma* spp. were not identified, while genetic material from 16 *Ehrlichia* spp. were detected. Three of the genetic materials clustered with *E. canis,* while eight were phylogenetically related to *E. chaffeensis*. Of the remaining five genetic materials, three clustered phylogenetically with *E. muris,* while two were closely related to the incompletely described *Ehrlichia* sp. UFMG-EV *minasensis*.

*Ehrlichia canis* can cause illness in dogs and other canids, which are thought to be the reservoir hosts of the pathogen. Human infections from *E. canis* have been reported, but the incidence is quite low. In Venezuela, chronic, asymptomatic infections by *E. canis* in human patients have been described, in addition to six clinical cases of ehrlichiosis [8, 23]. All of the patients with clinical cases had a fever, headache, and generalized body pain. In some patients, arthralgia, diarrhea and vomiting, malaise, nausea accompanied by body rash, and abdominal pain also occurred, while leukopenia was observed in one patient and anemia in another [8, 23]. The six patients were young and apparently healthy, and the *E. canis* strains obtained from them were indistinguishable from those found in dogs [8, 23]. *Ehrlichia canis* nucleic acids also have been detected from stored human blood samples in the U.S. Bouza-Mora et al. [24] recently reported the detection of a novel *E. canis* in blood samples collected from blood donors in Costa Rica. Although *E. canis* occurs worldwide, its presence and density varies with the geographic distribution of its tick vectors, like any other tick-borne bacterial pathogen. The presence of a novel *Ehrlichia* genotype suspected to be *E. canis* in dogs in South Africa has been reported by Allsopp and Allsopp [25]. In a study carried out by Williams et al. [26] on dogs and wild canids in Zambia, there was no reported detection of *E. canis* among the study population, while Matjila et al. [27] reported a 3% prevalence of *E. canis* in blood samples collected over a period of 7 years from dogs in South Africa. The natural tick host of *E. canis* is the brown dog tick, *R. sanguienius* s.l, which is widely distributed in South Africa. Here we report the genetic detection of *E. canis* from ticks collected from domesticated ruminants. We detected the genetic material of *E. canis* in four samples from *R. sanguineus* s.l. collected from cattle and sheep. Dogs are naturally infected with this pathogen, but its presence in ticks collected from cattle and sheep should not be unusual since dogs are companion pets of herdsmen. Moreover, it is possible for an infected questing tick of any age to attach itself to any available animal host; hence, finding *E. canis* in cattle and sheep is not unusual. One study of *E. canis* in South Africa reported a prevalence of 1% [27]; another study by Pretorius and Kelly [28] was based on the seroprevalence of *Ehrlichia* spp., which in most cases is not definitive due to lack of specificity of antibody reactions in distinguishing infections caused by *Ehrlichia* spp. The reports on *E. canis* detection in ticks collected from ruminants across the globe also is limited except for Zhang et al. [29], who reported the detection of *E. canis* in the blood of ruminants collected on five Caribbean Islands. On the African continent, Ndip et al. [13] reported a 16.3% prevalence of *E. canis* in dogs studied in Cameroon.

*Ehrlichia chaffeensis* is an emerging tick-borne pathogen known to cause illness in humans [22]. According to the Centers for Disease Control [30], ehrlichiosis was first documented as a disease in the U.S. in the late 1980s but became a reportable disease only in 1999. Since then, the number of ehrlichiosis cases due to *E. chaffeensis* has increased steadily both in the U.S. and across the globe [7]. *Ehrlichia chaffeensis* is responsible for the disease known as human monocytic ehrlichiosis (HME). White-tailed deer (*Odocoileus virginianus*) are the major reservoir host of *E. chaffeensis* in the U.S., although it has also been detected across the globe in other deer species, such as the spotted deer (*Cervus nippon*) in Japan and Korea and the marsh deer in Brazil, as well as in numerous other wild and domesticated animals [20, 21, 31]. Also, *E. chaffeensis* genetic materials have been detected by PCR in coyotes in the US and wild lemurs while antibodies to the bacteria have been reported in opossums, raccoons, rabbits and foxes [32–38].

Ehrlichiosis is most commonly detected in the Southeastern and South Central United States, an area corresponding to the natural habitat of *Amblyomma americanum,* the known vector of *E. chaffeensis* [30, 39–42]. Data on the true prevalence of *E. chaffeensis* in South Africa is limited. On the African continent, ehrlichiosis in humans caused by *E. chaffeensis* has been reported in Cameroonian patients by Ndip et al. [7], with a prevalence of 10% among 118 patients. *Amblyomma americanum*, the tick vector of *E. chaffeensis,* is found only in the U.S.; however, reports abound in the literature of the detection of *E. chaffeensis* DNA in other tick species, such as *Dermacentor variabilis, Ixodes pacificus, A. testudinarium, Haemaphysalis longicornis* and *H. yeni* [7, 43–47], suggesting that other vector agents do exist. Here, we report the detection of genetic materials from ticks that clustered phylogenetically close to *E. chaffeensis* sequences obtained from the GenBank in a phylogenetic tree. The genetic materials were detected in *A. hebraeum* collected from both cattle and sheep in two communities within the study areas, thus supporting reports that other ticks may be vectoring this pathogen across the globe.

In 2009, an *E. muris*-like (EML) pathogen was detected in four patients in Minnesota and Wisconsin in the U.S., thus implicating this bacterium, which is commonly found in rodents, in human infections [48–50]. *Ehrlichia muris* is capable of causing infections in humans characterized by fever, headache, nausea, vomiting and fatigue [51]. DNA from this bacterial pathogen also has been detected in the blood of deer and small rodents, with the latter having been suggested as the probable reservoir host [51] for *I. persulcatus,* which feeds on them as its tick vector [30]. The first description of *E. muris* was in rodents in Japan [49–52], where it also was associated with human infection first. Ravyn et al. [53] have also reported the identification of *E. muris* from *I. persulcatus* ticks in Russia, while Spitalská et al. [54] detected *E. muris* in *I. ricinus* in Slovakia, thus suggesting the possibility of the pathogen’s maintenance via rodent-feeding ticks. Three of the *Ehrlichia*-positive samples detected in this study contained genetic materials that clustered phylogenetically *with E. muris,* which to the best of our knowledge is being reported for the first time in South Africa. Also detected in this study were two sequences that clustered with the *Ehrlichia* sp. UFMG EV *minasensis*, a new genotype phylogenetically close to *E. canis* that was first identified in infected cattle and deer in Canada. *Ehrlichia* sp. UFMG EV *minasensis* also has been reported in *R. microplus* ticks and cattle in Brazil by Cabezas-Cruz et al. [10] and Carvalho et al. [11]. The pathogenic potential of this new *Ehrlichia* sp. is yet to be determined.

Reports on human ehrlichiosis in Africa is uncommon, as only Ndip et al. [7] have thus far characterized genetic material belonging to the bacteria in Cameroon. Another study on the seroprevalence of antibodies to *Ehrlichia* in blood samples collected across Africa showed that only two samples, one each from Mali and Mozambique, were positive [55]. The severity of ehrlichiosis depends on the individual immune status, as people with compromised immunity caused by HIV infection, immunosuppressive therapies or splenectomies develop more severe disease [30]. Because of the high HIV/AIDS prevalence in the Eastern Cape of South Africa, the incidence of ehrlichiosis could be high. However, a comprehensive study is yet to be conducted on this emerging disease. With a 2.3% prevalence of this emerging tick-borne pathogen, there is a good chance that human infections could be going undetected as they may be mistaken for the flu or flu-like infections. The short duration of this study poses one limitation to our findings; likewise, few sites were used and the number of ticks collected was low. Further studies are needed to redress these limitations.

## Conclusion

Infectious diseases do not respect international boundaries and there are very few systematic studies of tick-borne pathogens in ticks collected in South Africa. The prevalence of *Ehrlichia* spp. among ticks collected in this study was low and *Anaplasma* spp. was not detected in any samples tested. For a better understanding of the true situation, long-term surveillance of the prevalence of *Ehrlichia* and other tick-borne bacterial pathogens in South Africa that incorporates different climatic conditions and wider geographic areas is necessary in order to accurately determine the disease transmission risks associated with emerging tick-borne bacterial pathogens and the distribution of their vectors.

## Abbreviations

CME: Canine monocytic ehrlichiosis
HME: Human monocytic ehrlichiosis
